# Tuberculosis chemotherapy: current drug delivery approaches

**DOI:** 10.1186/1465-9921-7-118

**Published:** 2006-09-19

**Authors:** Lisa Claire du Toit, Viness Pillay, Michael Paul Danckwerts

**Affiliations:** 1 University of the Witwatersrand, Faculty of Health Sciences, Department of Pharmacy and Pharmacology, 7 York Road, Parktown, 2193, South Africa

## Abstract

Tuberculosis is a leading killer of young adults worldwide and the global scourge of multi-drug resistant tuberculosis is reaching epidemic proportions. It is endemic in most developing countries and resurgent in developed and developing countries with high rates of human immunodeficiency virus infection. This article reviews the current situation in terms of drug delivery approaches for tuberculosis chemotherapy. A number of novel implant-, microparticulate-, and various other carrier-based drug delivery systems incorporating the principal anti-tuberculosis agents have been fabricated that either target the site of tuberculosis infection or reduce the dosing frequency with the aim of improving patient outcomes. These developments in drug delivery represent attractive options with significant merit, however, there is a requisite to manufacture an oral system, which directly addresses issues of unacceptable rifampicin bioavailability in fixed-dose combinations. This is fostered by the need to deliver medications to patients more efficiently and with fewer side effects, especially in developing countries. The fabrication of a polymeric once-daily oral multiparticulate fixed-dose combination of the principal anti-tuberculosis drugs, which attains segregated delivery of rifampicin and isoniazid for improved rifampicin bioavailability, could be a step in the right direction in addressing issues of treatment failure due to patient non-compliance.

## Background

Tuberculosis (TB), a ubiquitous, highly contagious chronic granulomatous bacterial infection, is still a leading killer of young adults worldwide. TB has returned with a new face and the global scourge of multi-drug resistant TB (MDR TB) is reaching epidemic proportions.

Nearly one-third of the global population – two billion people – is infected with *Mycobacterium tuberculosis *(*M. tuberculosis*), more than eight million people develop active TB every year, and approximately two million die annually (World Health Organization, 2003). TB is the world's second most common cause of death from infectious disease, after acquired immunodefiency syndrome (AIDS) [1]. It is endemic in most developing countries and resurgent in developed and developing countries with high rates of human immunodeficiency virus (HIV) infection. With particular reference to Africa, the increase in TB incidence is strongly associated with the prevalence of HIV infection: rates of HIV infection among TB patients are correspondingly high, exceeding 60% in South Africa, Botswana, Zambia, and Zimbabwe [2].

Mortality rates of TB range from 50 to 80% in untreated smear-positive individuals to 30% with inconsistent control programmes and drop to lower than 5% when directly observed therapy (DOT) and active TB control programmes are instituted [2].

**Figure 1:**
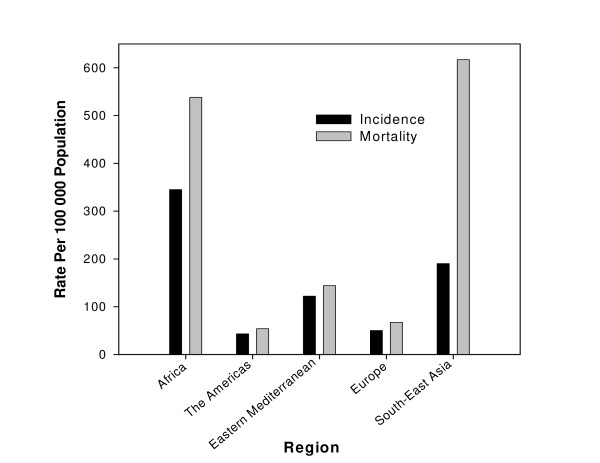
**Estimated TB incidence and mortality in 2003**. Data extracted from WHO Tuberculosis data sheet [3].

The TB incidence (number of new cases arising each year) and mortality in each of the WHO regions is depicted in Figure 1. The incidence of all forms of TB, the incidence of infectious cases, and mortality are represented as the rate per 100 000 population [3]. The majority of cases (5–6 million) are in people aged 15–49 years. The largest number of cases occurs in the South-East Asia Region, which accounts for 33% of incident cases globally. However in 2003, the estimated incidence per capita in sub-Saharan Africa was nearly twice that of the South-East Asia, at 290 to 350 cases per 100 000 population [1,3].

*M. tuberculosis *is a highly contagious, airborne, slow-growing, Gram-positive aerobic rod-shaped acid-fast bacillus. The cell wall has high lipid content and allows the bacteria to survive within macrophages. It also provides the organism with a resistant barrier to many common drugs [4,5].

**Figure 2:**
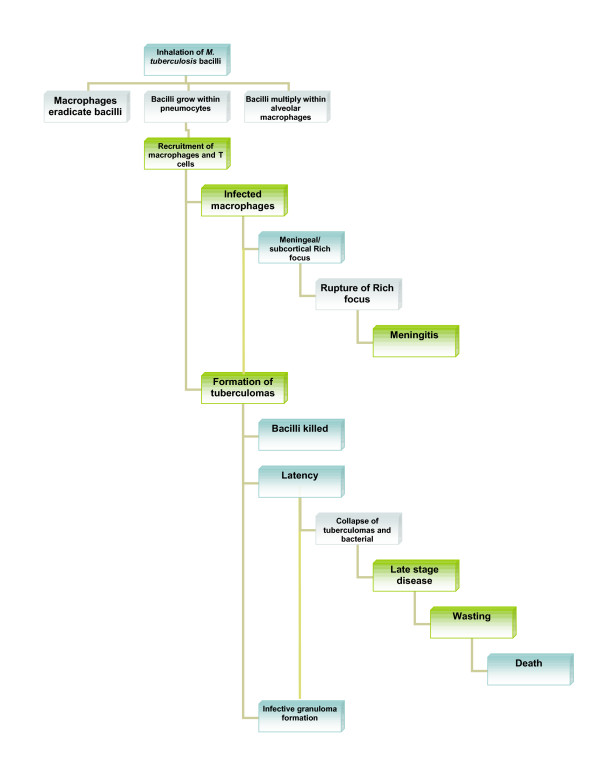
Pathogenesis of TB.

Man is the primary host for *M. tuberculosis*. Infection is spread via airborne dissemination of aerosolised bacteria-containing droplet nuclei of 1–5 μm in diameter that carry *M. tuberculosis *droplets from an individual with infectious TB disease to an uninfected individual. The infectious droplet nuclei are inhaled and lodge in the alveoli in the distal airways. *M tuberculosis *is then taken up by alveolar macrophages, initiating a cascade of events that results in either successful containment of the infection or progression to active disease (primary progressive TB). Risk of development of active disease varies according to time since infection, age, and host immunity, however, the lifetime risk of disease for a newly infected young child has been estimated at 10% [1,6,7]. Schematic representation of the progression of TB is shown in Figure 2.

This article seeks to review the current situation in terms of drug delivery approaches for TB chemotherapy. In so doing, the need for the development of a novel oral system that could overcome the bioavailability concerns of currently available fixed-dose anti-TB drug combinations in addition to assisting in achieving improved patient compliance with the existing regimen, will be addressed.

## Currect anti-tuberculosis chemotherapy

Since the control measures for TB such as Bacillus Calmette-Guérin (BCG) vaccination and chemoprophylaxis appear to be unsatisfactory, treatment with anti-tubercular (anti-TB) drugs becomes the only option available. The goals of treatment are to ensure cure without relapse, to prevent death, to impede transmission, and to prevent the emergence of drug resistance. Long-term treatment with a combination of drugs is required [8]. Treatment of active TB with a single drug should never be attempted, and a single drug should never be added to a failing regimen, the result being development of MDR TB [9]. As suggested by WHO [10], treatment of TB and drug resistant cases requires multi-drug therapy, comprising:

1. An initial intensive phase of rifampicin (RIF), isoniazid (INH), pyrazinamide (PYZ), and ethambutol (ETB) daily for 2 months.

2. A continuation phase of RIF and INH for a further 4 months, either daily or 3 times per week, to be administered as advised in Table 1.

**Table 1:** Regimen 1 – for treatment of new smear positive adult patients

**Intensive Phase – 2 months**	**Under 50 kg**	**Over 50 kg**
RIF/INH/PYZ/ETB		
Combination tablet 120/60/300/200 mg daily, 5 days per week	4 tablets	5 tablets

**Continuation phase – 4 months**	**Under 50 kg**	**Over 50 kg**

RIF/INH		
Combination tablet 150/100 mg	3 tablets	
Combination tablet 300/150 mg	-	2 tablets

Data extracted from Gibbon [11]

**Figure 3:**
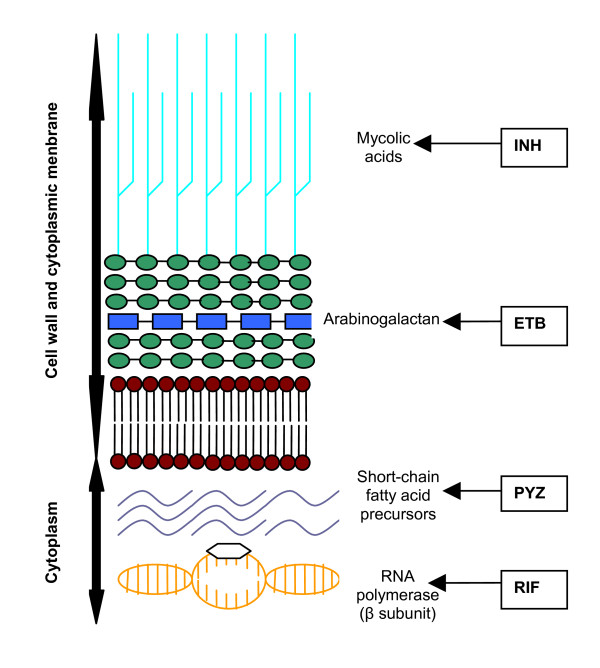
**Sites of action of the principal anti-TB drugs**. Adapted in part from Rattan et al.; Parsons et al.; Somoskovi et al. [16,17,18].

INH eradicates most of the rapidly replicating bacilli in the first 2 weeks of treatment, together with streptomycin and ETB. Thereafter, RIF and PYZ have an important role in the sterilisation of lesions by eradicating organisms; these two drugs are crucial for successful 6-month treatment regimens. RIF kills low or non-replicating organisms and the high sterilising effect of PYZ serves to act on semidormant bacilli not affected by any other anti-TB agents in sites hostile to the penetration and action of the other drugs [12,13]. INH and RIF, the two most potent anti-TB drugs, kill more than 99% of tubercular bacilli within 2 months of initiation of therapy [14,15]. Using these drugs in conjunction with each other reduces anti-TB therapy from 18 months to 6 months. The sites of action of these principle anti-TB agents are schematically illustrated in Figure 3, as delineated by Rattan et al., Parsons et al., and Somoskovi et al. [16-18]. The emergence of strains resistant to either of these drugs causes major concern, as treatment is then deferred to drugs that are less effective, have more toxic side effects, and result in higher death rates, especially among HIV-infected persons [16]. The current armamentarium of drugs available for the treatment of TB, their mechanism of action, and activity, have been reviewed by numerous authors [4,5,19-40] and appear in Table 2.

**Table 2:** Classes of anti-TB drugs

***Agent***	***Mechanism of Action***	***Activity Against M. tubercolosis***
**First-line agents**		
Rifampicin (RIF)	Inhibits bacterial RNA synthesis by binding to the β subunit of bacterial DNA-dependent RNA-polymerase (DDRP) Inhibition of DDRP leads to blocking of the initiation chain formation in RNA synthesis. One of the most effective antituberculosis agents available and is bactericidal for intra- and extra-cellular bacteria [4,19].	RIF inhibits susceptible organisms at concentrations of less than 1 μg/mL [4].
Isoniazid (INH)	Most active drug for the treatment of TB caused by susceptibe strains. Is a pro-drug activated by katG, which exerts its lethal effect through inhibition of synthesis of mycolic acids, an essential component of mycobacterial cell walls, through formation of a covalent complex with an acyl carrier protein (AcpM) and KasA, a beta-ktoacyl carrier protein synthetase [4,5]	INH inhibits tubercle bacilli at a concentration of 0.2 μg/mL [4].
Pyrazinamide (PYZ)	Converted to the active pyrazanoic acid (encoded by pncA) by pyrazinamidase in susceptible organisms. Pyrazanoic acid lowers pH in the immediate surroundings of *M. tuberculosis *– organism is unable to grow. May also function as an antimetabolite of nicotinamide and interfere with the synthesis of NAD, inhibiting the synthesis of short-chain, fatty-acid precursors [4,5]	Inhibits *M. tuberculosis *and other mycobacteria at concentrations of 20 μg/mL [4].
Ethambutol (ETB)	Inhibits mycobacterial arabinosyl transferases (encoded by the embCAB operon) involved in the polymerization of D-arabinofuranose to arabinoglycan, an essential cell wall component [4,5].	Ethambutol is generally bacteriostatic, but at high doses (25 mg/kg) can be bactericidal [20]. Inhibits susceptible strains of M. tuberculosis at concentrations of 1–5 μg/mL [4].

**Aminoglycosides (injectable)** Streptomycin, kanamycin, amikacin, capreomycin	The aminoglycosides are irreversible inhibitors of protein synthesis through binding to specific 30S-subunit ribosomal proteins [4].	Bactericidal. *In vitro *and *in vivo *clinical data support use [21,22,23].

**Fluoroquinolones** Ciprofloxacin, ofloxacin, levofloxacin, moxifloxacin, gatifloxacin, sparfloxacin	Inhibit bacterial DNA synthesis through inhibition of bacterial topoisomerase II (DNA gyrase) and topoisomerase IV, which are responsible for the relaxation of supercoiled DNA and the separation of replicated chromosomal DNA, respectively [4].	Bactericidal, broad spectrum antibacterials [5]. In-vitro and in-vivo clinical data support use [24,25]. Ciprofloxacin and levofloxacin inhibit strains of *M. tuberculosis *at concentrations of less than 2 μg/ml. Newer agents (moxifloxacin, gatifloxacin, sparfloxacin) have lower minimum inhibitory concentrations [4,26,27].

**Bacteriostatic second-line drugs**		
Ethionamide	Chemically related to INH, converted via oxidation to ethionamide sulfoxide, blocks the synthesis of mycolic acids [4,5].	Inhibits most tubercle bacilli at concentrations of 2.5 μg/mL or less [4].
Cycloserine	Structural analogue of D-alanine, inhibits incorporation of D-alanine into peptidoglycan pentapeptide through inhibition of alanine racemase [4].	Inhibits strains of *M. tuberculosis *at concentratins of 15–20 μg/mL [4].
P-aminosalicylic acid	Anti-metabolite interfering with incorporation of para-aminobenzoic acid into folic acid – folate synthesis antagonist [4,5].	Inhibits tubercle bacilli at concentrations of 1–5 μg/mL [4].

**Other drugs**	Potentially useful agents with conflicting animal or clinical evidence or agents with unclear efficacy because of possible cross-resistance.	
Clofazimine	Unknown, but may involve DNA binding [4]. Possesses direct antimycobacterial and immunosuppressive properties [4,5]	Bacteriostatic *in vitro*. MIC 90 <1.0 mg in vitro [28]. Apt concentrations attainable *in vivo*, particularly in macrophages [29,30].
Amoxicillin/clavulanic acid	Amoxicillin (a penicillin) inhibits cell wall synthesis. Clavulinic acid is a beta-lactamase inhibitor	β lactams in combination with beta lactamase inhibitors bactericidal *in vitro *[31,32,33].
Clarithromycin	Inhibition of protein synthesis via binding to 50S ribosomal RNA as aminoacyl translocation reactions and the formation of initiation complexes are blocked [4,5].	Although *in vitro *antimycobacterial properties reported, data from animal and *in vivo *studies conflicting [34,35,36,37].
Rifabutin	Activity is similar to that of rifampicin. Inhibits bacterial RNA synthesis by binding strongly to the β subunit of bacterial DNA-dependent RNA-polymerase [4].	May be useful against some isolates of MDR TB (resistant to RIF *in vitro *but sensitive to rifabutin) [38]. Effective in prevention and treatment of disseminated atypical mycobacterial infection in AIDS patients with CD4 counts <50 [4].
Thiacetazone	Not clearly elucidated.	*In vivo *and *in vitro *evidence of bacteriostatic activity. Cross-resistance frequently seen between thiacetazone and both INH and ethionamide [39,40].

TB is treated with a multi-drug regimen, and is thus exceptionally vulnerable to incidences of side effects, unsatisfactory patient compliance and slow improvement of patients [41]. Therefore, despite the availability of these highly effective treatments for TB, cure rates remain low, as commercial anti-TB formulations are inconvenient to administer and patients do not take the prescribed medications with sufficient regularity and duration to achieve a cure [42]. Patients have to consume a large number of tablets (up to eight at one time), which is a common cause for non-compliance. It can be anticipated that non-optimal application of these short course regimens will result in the deterioration of their therapeutic potential, an escalation in the mortality rate and increased risk of developing acquired drug resistance [3,43,44]. Resistance of *M. tuberculosis *to anti-TB agents is a worldwide problem in both immunocompetent and HIV-infected populations [45,46].

## Novel drug delivery systems for the treatment of TB

Chemotherapy of TB is complicated by the need of multi-drug regimens that need to be administered over long periods. Poor patient compliance is the single most common reason for chemotherapy failure in TB [41]. To minimise toxicity and improve patients' compliance, extensive progressive efforts have been made to develop various implant-, microparticulate-, and various other carrier-based drug delivery systems to either target the site of *M. tuberculosis *infection or reduce the dosing frequency, which forms an important therapeutic strategy to improve patient outcomes [47,48]. The systems under discussion employ either biodegradable polymers or systems requiring removal after use, and can release the drug either by membrane or matrix-controlled diffusion.

Recent trends in controlled drug delivery have seen microencapsulation of pharmaceutical substances in biodegradable polymers as an emerging technology. Carrier or delivery systems such as liposomes and microspheres have been developed for the sustained delivery of anti-TB drugs and have demonstrated better chemotherapeutic efficacy when investigated in animal models (e.g. mice) [47]. Anti-TB drugs have been successfully entrapped and delivered in biodegradable polymers such as poly (DL-lactide-co-glycolide) (PLG), which are biocompatible and release drug in a controlled manner at therapeutic levels [49]. Dutt and Khuller [50,51] have entrapped INH and RIF in PLG polymers. When injected subcutaneously as a single dose, the microparticles, having a diameter ranging from 11.75 μm to 71.95 μm, provided sustained release of drugs over 6–7 weeks when tested in mice [50]. The authors previously observed that particles with a size range >10 μm remained at the site of injection forming a depot. The entrapped contents of the microparticles were gradually released by diffusion through the polymeric particles. Such depots can show release profiles extending over several months culminating in degradation of the entire polymeric device.

However, these formulations have to be injected either subcutaneously or intravenously, and the pain and discomfort associated with these routes of administration, in general, is often not acceptable. Hence, there is a continuous need to develop an oral drug delivery system that is convenient for patients [41].

Amidst these concerns, Ain et al. [52] reported the pharmacokinetics of PLG encapsulated anti-TB drugs; orally administered either individually or in combination in mice. A study conducted by Pandey et al. [48] reported the formulation of three frontline anti-TB drugs, i.e. RIF, INH and PYZ encapsulated in PLG nanoparticles. On oral administration of drug-loaded nanoparticles to *M. tuberculosis*-infected mice at every 10th day, no tubercle bacilli could be detected in the tissues after 5 oral doses of treatment. Therefore, oral nanoparticle-based anti-TB drug therapy can allow for a reduction in dosing frequency for better management of TB. Prabakaran [41] developed an osmotically regulated capsular multi-drug oral delivery system comprising asymmetric membrane coating- and dense semipermeable membrane coating-capsular systems for the simultaneous controlled administration of RIF and INH for the treatment of TB. This was in an attempt to reduce the problems associated with multi-drug therapy. The modified asymmetric system provided satisfactory sustained release of RIF and INH, with an initial burst release that may be sufficient to achieve minimum effective concentration in blood. Thereafter, the system provided the release of the drugs in a near zero order rate – an ideal release profile for controlled drug delivery. In turn, this would improve the safety profile of the drugs and enhance the activity duration of drugs exhibiting short half-lives. The once daily system is optimal, and could potentially enhance patient compliance.

In addition to these combinations, the past several years have seen the development of a number of RIF-only controlled release formulations for the improvement of the clinical efficacy of the drug and patient compliance [53-62].

Further attempts to solve the problems inherent in multi-drug therapy have included the development of biodegradable polymeric micro- or nanoparticulate carrier systems to target alveolar macrophages that harbour *M. tuberculosis *[51,63-67]. In the case of pulmonary TB, delivering the drug directly to the site of infection through inhalation of an aerosolised delivery system has the inherent advantages of bypassing first-pass metabolism and maintaining local therapeutically effective concentrations with decreased systemic side effects [68]. Because *M. tuberculosis *is known to infect alveolar macrophages and affect the pathogenesis of TB, there have been renewed interests in targeting of anti-TB drugs to these cells. Despite the success of these systems in targeting and providing sustained release of anti-TB drugs to alveolar macrophages, the methods used to generate particles in these studies vary in their capability for the production of reproducible particles with the optimal size for inhalation therapy (i.e. <5 μm). Barrow et al. [64] formulated RIF-loaded microspheres using the method of solvent evaporation, aiming to maintain a size of 1-to 10-μm. Only the size distributions of two formulations were reported, being 3 to 4 μm, and distribution demonstrated a Gaussian curve. Dutt and Khuller [51] encapsulated INH and RIF into hardened PLG microparticles by a double emulsification solvent evaporation procedure, and these had a resultant volume mean diameter of 11.75 μm for INH microparticles and 11.64 μm for RIF microparticles. These are currently undergoing Phase I trials. Sharma et al. [66] incorporated both INH and RIF into PLG microspheres using a combination of solvent extraction and evaporation, but these particles had a mean diameter of 6.214 μm and only 38% of the microspheres fell in the size range of 0.5–3 μm. Suarez et al. [68,69] attained the airway delivery of RIF microparticles having volume median diameters of 2.76 ± 1.57 μm. O'Hara and Hickey [70] succeeded in obtaining RIF-loaded PLG particles with median diameters by volume of 2.76 μm and 3.45 μm by spray drying and solvent evaporation respectively. Zhou et al. [68] did achieve the formulation of spherical microparticles between 1 and 3 μm in diameter. The microparticles, prepared by the precipitation with a compressed antisolvent process, were evaluated for their potential in targeting an ionizable prodrug of INH, isoniazid methanesulfonate (INHMS), for sustained delivery of INH to alveolar macrophages.

Most recently Zahoor et al. [71,72] undertook pharmacokinetic and chemotherapeutic studies with aerosolised alginate nanoparticles encapsulating INH, RIF and PZA and RIF, INH, PYZ, ETB. The nanoparticles were prepared by cation-induced gelification of alginate and were 235.5 ± 0 nm in size, with drug encapsulation efficiencies of 70–90% for INH and PZA and 80–90% for RIF and 88–95% for EMB. The majority of particles (80.5%) were in the respirable range, with a mass median aerodynamic diameter of 1.1 ± 0.4 μm and geometric standard deviation of 1.71 ± 0.1 μm. The chemotherapeutic efficacy of three doses of drug-loaded alginate nanoparticles nebulised 15 days apart was comparable with 45 daily doses of oral free drugs. Thus, inhalable alginate nanoparticles could potentially serve as an ideal carrier for the controlled release of anti-TB drugs. Clinical trials are envisaged in the future for evaluation of this system before use in humans.

Pandey and Khuller [73] evaluated the chemotherapeutic potential of nebulised solid lipid nanoparticles (SLNs) incorporating RIF, INH and PYZ against experimental TB. SLNs are nanocrystalline suspensions in water, prepared from lipids, which are solid at room temperature. The SLNs, prepared by the emulsion solvent diffusion technique, possessed a favourable mass median aerodynamic diameter suitable for bronchoalveolar drug delivery. Following a single nebulisation to guinea pigs, therapeutic drug concentrations were maintained in the plasma for 5 days and in the organs for 7 days whereas free drugs were cleared after 1–2 days. Vyas [74] formulated aerosolised liposomes incorporating RIF via a cast-film method employing egg phosphatidylcholine- and cholesterol-based liposomes. Liposomes coated with alveolar macrophage-specific ligands demonstrated preferential accumulation in alveolar macrophages, maintaining high concentrations of RIF in the lungs even after 24 hours.

In another approach to solve the predicament of poor patient compliance, depot-delivery of anti-TB drugs has been investigated. Studies have demonstrated that a single implant of INH in polylactic-co-glycolic acid (PLGA) copolymer could ensure sustained levels of free INH for a period of up to 8 weeks following implantation in rabbits [49]. Gangadharam, et al. [74] have also investigated the chemotherapy of TB in mice using single implants of INH and PYZ. Such devices, however, inherently suffer from the disadvantages of immobilisation at the implanatation site and surgical requirements for implantation.

A number of the aforementioned developments in drug delivery represent attractive options with significant merit, and the pertinent points regarding each exemplary investigation are summarised in Table 3; however, the need to develop an oral drug delivery system with improved patient acceptance is affirmed by the accelerated pace of oral drug delivery system development fostered by the need to deliver medications to patients more efficiently and with fewer side effects, especially in developing countries where controlled-delivery implants and injectables could be too expensive.

**Table 3:** Synopsis of Novel Anti-TB Drug Delivery Systems

***Drug***	***Delivery System and Polymer Employed***	***ROA***	***Preparatory Methods***	***Characterisation Studies and System Suitability***	***Reference***
INH	Porous, non-porous and hardened microparticles employing PLG	SC injection	Double emulsification solvent evaporation	Size: Mean volume diameters were: 62.11 μm, 71.95 μm and 11.75 μm for porous, non-porous and hardened microparticles, respectively. *In vitro *studies: Sustained release of INH up to 6 days from non-porous microparticles. Porous microparticles released INH over 3 days. Hardened PLG microparticles sustained release of INH for up to 7 weeks *In vivo*disposition studies (*in mice*): Porous and non-porous microparticles released INH in plasma for up to 2 days. Hardened PLG microparticles sustained release of INH for up to 7 weeks. Concentrations of INH obtained were higher than the MIC of INH.	[50]
RIF, INH, PYZ, ETB	Microparticles employing PLG	Oral, singly or in combi-nation	Double emulsification solvent evaporation	DEE: 8–10% for PZA; 10–11% for INH and 12–18% for RIF. Size: Diameters were 1.11 μm for INH, 1.40 μm for RIF and 2.20 μm for PZA microparticles. *In vitro *studies: Entrapped drugs were released in a sustained manner. In the intestinal fluid drug release was obtained for up to 20 days *In vivo *studies: Entrapped drugs remained in circulation up to 72 h as compared to free drugs (eliminated within 24 h). Level of PLG encapsulated INH was found to be higher than its MIC value (0.1 μg/ml). Pharmacokinetic analysis (*PLG encapsulated drugs and free drugs*): Increased *C*_max_; AUC_o-α_; *t*_1/2 _(a) and *t*_1/2 _(e) when drug were given entrapped in PLG microparticles indicated the potential of PLG for effective treatment of TB	[52]
RIF, INH, PYZ	Nanoparticles employing PLG	Oral	Multiple emulsion technique	Size: Majority (>80%) in the size range of 186–290 nm, polydispersity index of 0.38 ± 0.04 DEE: 56.9 ± 2.7% for RIF, 66.3 ± 5.8% for INH and 68 ± 5.6% for PZA. Drug loading: 570 to 680 mg drug per gram of polymer. *In vitro *studies: drug release profile in PBS showed an initial (up to 48 h) burst release followed by a negligible release of either drug up to 6 weeks. *In vivo *studies (*experimental infection and chemotherapy*): following oral administration of drug-loaded nanoparticles to *M. tuberculosis*-infected mice at every 10th day – no tubercle bacilli could be detected in the tissues after 5 oral doses of treatment	[48]
RIF, INH	Osmotically regulated capsular multi-drug oral delivery system employing HPMC and NaCMC	Oral	Phase inversion process – precipitation of membrane structure on a stainless steel mould pin	SEM: porous structure of the membranes was evident. *In vitro *studies: sustained release of RIF and INH, with initial burst release, which may be sufficient to achieve MIC in blood. Thereafter, the system sustained the release of the drugs in a near zero order rate *In vitro *release kinetics: first order kinetics. Statistical analysis of release rate data – modified asymmetric system the preferred system.	[41]
INH, RIF	Microparticles employing PLG	SC, Inhaled	Double emulsification solvent evaporation	Size: Volume mean diameters of 11.75 μm (INH-loaded) and 11.64 μm (RIF-loaded) DEE: 10–11% (INH-loaded) and 12–14% (RIF-loaded) *In vivo*-combination drug disposition studies and experimental infection and chemotherapy studies: single dose of PLG microparticles – sustained release of INH and RIF for up to 7 and 6 weeks, respectively. Free drugs (in combination) injected in the same doses were detectable *in vivo *up to 24 h only. One dose of PLG microparticles cleared bacteria more effectively from lungs and liver in experimental murine model of TB (compared with a daily administration of the free drugs) Phase I trials	[51]
RIF	Microspheres employing PLG	Inhaled/aerosol	Solvent evaporation	Size: reported for 2 formulations – 3 to 4 μm, and distribution demonstrated a Gaussian curve. *In vitro *studies: best *in vitro *release patterns, resulted in 21 and 12% cumulative *in vitro *drug release, respectively, after 6 days Release in monocytic cell lines (*murine J774 and the human Mono Mac 6*): Bioassay assessment of cell culture supernatants from monocyte cell lines – release of RIF during a 7-day experimental period. Treatment of *M. tuberculosis *H37Rv-infected monocyte cell lines with RIF-loaded microspheres resulted in a significant decrease in numbers of CFU at 7 days following initial infection	[64]
INH, RIF	Microspheres employing PLG	Inhaled	Combination of solvent extraction and evaporation	Size: mean diameter of 6.214 μm and only 38% of the microspheres fell in the size range of 0.5–3 μm *In vivo *studies: Microspheres were tested for uptake by murine macrophages in culture and resultant intracellular drug concentrations. The extent of microparticle delivery *in vivo *was examined by flow-cytometry. Drug concentrations (blood and alveolar macrophages) estimated after oral, vascular, intratracheal, and inhalation administration. Large numbers of particles delivered to the bronchiopulmonary system through a 2 min exposure to fluidized particles. The intracellular drug concentrations resulting from vascular delivery of soluble drugs were lower than those resulting from particle inhalation.	[66]
RIF	Microparticles employing PLGA	Inhaled	Spray drying	Size: Volume median diameters (VMD) and geometric standard deviations (S.D.) were [VMD (μm)/geometric S.D.]: RIF-PLGA, 2.76/1.57; PLGA, 2.87/1.45; and RIF alone, 3.83/1.75. *In vivo *studies: Alveolar macrophage *M. tuberculosis*(H37Rv)-infected guinea pig model was used to screen for targeted delivery to the lungs by insufflation (with lactose excipient) or nebulisation RIF-PLGA microspheres. Animals treated with single and double doses of RIF-PLGA microspheres – reduced numbers of viable bacteria, inflammation and lung damage compared with RIF-only treated animals 28 days post-infection. Two doses of RIF-PLGA – reduced splenic enlargement.	[69]
RIF	Microparticles employing PLGA	Inhaled	Solvent evaporation and spray drying	Morphology: Spray dried RIF-loaded PLGA microparticles – shriveled morphology, spherical particles produced by solvent evaporation. Size: Median diameters by volume were 3.45 μm (solvent evaporation) and 2.76 μm (spray dried) DEE: 20% (solvent evaporation) and 30% (spray dried) Particles are being evaluated in an animal model of TB.	[70]
Ionizable prodrug of INH, INHMS	Spherical microparticles employing PLA	Inhaled	Precipitation with a compressed antisolvent process	Drug loading efficiency: 93 to 152% Size: Aerodynamic diameters ranged from 1 to 3 μm *In vitro *studies: Release profiles displayed two phases of drug release that were characterised by an initial burst effect, followed by a period of slower release *In vivo *studies (*drug accumulation in cultured rat alveolar macrophages*): Liquid chromatographic tandem mass spectrometric (LC-MS/MS) assay developed detected high level of INH in NR8383 (rat AM cell line) following exposure to drug-loaded microparticles. Compared INH levels in lavaged bronchoalveolar macrophages by LC-MS/MS after Sprague-Dawley rats administered either INHMS in PLA microparticles by intra-tracheal instillation or INH solution by gavage or intra-tracheal instillation – sustained delivery of INH to alveolar macrophages. Reduction in the blood levels of acetylisoniazid (AcINH), a major and potential toxic metabolite of INH.	[68]
INH, RIF, PZA and RIF, INH, PYZ, ETB	Nanoparticles employing alginate	Inhaled	Cation-induced gelification of alginate	Size: 235.5 ± 0 nm in size, with majority of particles (80.5%) were in the respirable range, with mass median aerodynamic diameter of 1.1 ± 0.4 μm and geometric standard deviation of 1.71 ± 0.1 μm. DEE: 70–90% for INH and PZA, 80–90% for RIF and 88–95% for ETB. *In vivo *studies (*disposition studies and chemotherapeutic studies*): The formulation was orally administered to mice at two dose levels. A comparison was made in mice receiving free drugs at equivalent doses. Relative bioavailabilities of drugs encapsulated in alginate nanoparticles significantly higher compared with oral free drugs. Drug levels were maintained at or above the MIC90 post nebulisation until Day 15 in organs (lungs, liver and spleen) after administration of encapsulated drugs, whilst free drugs stayed at or above the MIC90 up to Day 1 only irrespective of dose. Clinical trials envisaged in the future	[71,72]
RIF, INH and PYZ	Nebulised SLNs prepared from nanocrystalline lipid suspensions in water	Inhaled	Emulsion solvent diffusion technique	Size: favourable mass median aerodynamic diameter suitable for bronchoalveolar drug delivery *In vivo *studies: Therapeutic experimental TB drug concentrations were maintained in the plasma for 5 days and in the organs for 7 days whereas free drugs were cleared by 1–2 days	[73]
RIF	Aerosolised liposomes formulated using Egg PC-and Chol-based liposomes	Inhaled	Neutral liposomes were prepared by cast film method	Modification: Imparted negative charge (DCP) or by coating them with alveolar macrophage-specific ligands (MBSA and *O*-SAP). Size: neutral and negatively charged liposomes composed of PC:Chol:DCP had average vesicle size of 2.32 ± 0.48 μm and 2.50 ± 0.54 μm, respectively. MBSA-coated liposomes size: 3.64 ± 0.65 μm, *O*-SAP-coated vesicles size: 3.85 ± 0.59 μm. DEE: 47.4 ± 2.7% *In vivo *studies: Percent viability of *Mycobacterium smegmatis *inside macrophages (*in vitro*) after administration of drug (*in vivo*) was 7–11% (ligand-anchored liposomal aerosols), 45.7 and 31.6% in case of plain drug and plain neutral liposomal aerosol (based on PC:Chol)-treated macrophages. Preferential accumulation of MBSA- and *O*-SAP-coated formulations in alveolar macrophages. Drug was estimated in the lung in high concentration (even after 24 h).	[74]
INH	Implant prepared from PLGA	Depot	PLGA polymer rods	*In vivo *studies: Rods implanted in the back of rabbits under anaesthesia. Concentrations of INH and acetylisoniazid in serum and urine determined by HPLC. Concentrations of INH ≥ 0.2 μg/ml were found both in serum and urine up to 63 days after implant. Urine specimen obtained at 6 weeks after implant inhibited the growth of *M. tuberculosis in vitro *measured by the radiometric (Bactec) method.	[49]
INH, PYZ	Single implants prepared from PLGA	Depot	Depot drug preparation	*In vivo *studies: 3 times the daily dose of PYZ contained in single PLGA polymer implant – no burst levels of the drug evident after administration – sustained levels up to 54 days. Chemotherapeutic activity (investigated in mice) of the single PLGA polymer implants similar to standard oral treatment with the two drugs given daily for 8 weeks, determined by mortality and CFU counts of tubercle bacilli from lungs and spleen.	[75]

Chol = cholesterol, DCP = dicetylphosphate, DEE = drug entrapment efficiency, HPLC = high performance liquid chromatography, HPMC = hydroxypropylmethylcellulose, MIC = minimum inhibitory concentration, NaCMC = sodium carboxymethylcellulose, PBS = phosphate-buffered saline, PC = phosphatidylcholine, MBSA = maleylated bovine serum albumin, and *O*-SAP = *O*-steroyl amylopectin, ROA = route of administration, SC = subcutaneous, SLNs = solid lipid nanoparticles

## Rifampicin bioavailability concerns

The WHO and the International Union Against Tuberculosis and Lung Disease (IUATLD) encourage use of Fixed Dose Combination (FDC) formulations – this evolved from the fact that TB always requires multi-drug treatment [76]. Patients should thus be given FDC adjusted for body weight whenever self-administration of anti-TB drugs is permitted [3]. A FDC – which is a combination of two or more first-line anti-TB drugs in a single formulation at a fixed proportion – prevents monotherapy; and it is expected that this will reduce the emergence of MDR TB; simplify treatment, and thus minimize prescription error and increase patient and doctor compliance; simplify drug stock management, shipping and distribution; and reduce the risk of misuse of RIF for conditions other than TB [10].

Of dire concern, however, is the issue of unacceptable RIF bioavailability in a number of FDC anti-TB formulations [77,78]. The decomposition of RIF has varied from 8.5 to 50% in the acidic environment of the stomach in the time range corresponding to the gastric residence time for most dosage forms in humans (≈15 minutes to 105 ± 45 minutes) [79,80]. However, the gastric-emptying time for some single-unit dosage forms may reach 6 hours [81]. The use of substandard FDC will ultimately result in drug resistant TB and treatment failure [77]. The factors proposed for this variation in the bioavailability of RIF from different FDC formulations include the particle size and crystalline form of the drug, manufacturing process and the excipients employed [82,83]. The effect of these factors, however, has not been convincingly explained in previous studies. RIF is known to undergo hydrolysis in acidic medium to the insoluble 3-formyl rifamycin SV (3 FRSV). INH accelerates degradation of RIF into this poorly absorbed derivative (3 FRSV) in the acidic environment of the stomach via reversible formation of the isonicotinyl hydrazone of 3-FRSV with INH [80,84,85]. Shishoo et al. [80] has indicated that RIF in the presence of INH as a FDC, may undergo greater decomposition in the acidic conditions of the stomach, as compared to when RIF is administered (orally) alone. Thus, less RIF will be available for absorption from FDCs as compared to RIF administered as a separate formulation. This will be reflected in the poor bioavailability from the former formulation. There is thus an urgent need to modify or segregate the FDC formulation in such a way that RIF and INH are not released simultaneously in the stomach. Alternatively both drugs need to be administered separately after an interval corresponding to average gastric residence time, which is somewhat unpredictable due to high intra- and inter-subject variability [79,80,88].

**Figure 4:**
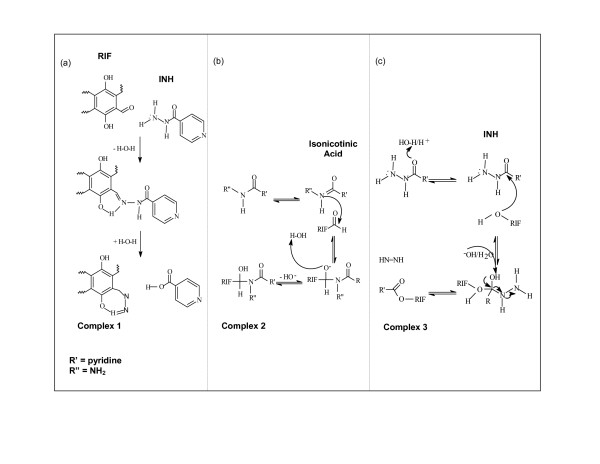
Proposed mechanisms for interaction between RIF and INH: (a) Schiff's Reaction of RIF and INH, (b) Carbonyl Condensation of RIF and INH, (c) Fischer's Esterification Reaction between RIF and INH.

Fairly recently, Chen [42] proposed a mechanism for this apparent degradation of RIF. 3-FRSV and INH could possibly undergo Schiff's reaction to form a complex (Figure 4(a)). The carbonyl groups and amine groups may rearrange to yield an iminium ion. The C-4 hydroxy group enhances the complex formation by possibly forming a hydrogen bond with the hydrogen atom attached to the nitrogen. This is a basic requirement of the Schiff's reaction. In addition, carboxylic acids and alcohols can also undergo carbonyl condensation reactions [89]. INH could react with RIF in this manner, which could account for the instability of RIF when present together with INH (Figure 4(b)). This interaction could also occur between RIF and PYZ, however, it has frequently been observed that INH caused further RIF stability reduction compared to PYZ. The reason for this could be due to the fact that the carboxylic acids and alcohol further undergo Fischer's esterification [89]. The hydroxyl groups of RIF are readily able to react with the aqueous carboxylic acid degradants yielded by INH and PYZ to form an ester (Figure 4(c)). However, as PYZ lacks the electron-withdrawing group, such as the secondary nitrogen found on the hydrazide group of INH, there is less tendency for this reaction to be expected between PYZ and RIF.

Permeability studies have demonstrated that RIF is well absorbed from the stomach due to its solubility, which has been shown to be maximal between pH 1–2. INH, although demonstrating solubility in the gastric environment, is comparatively well absorbed from all three segments of the small intestine. RIF and INH thus exhibit regional specific permeability, and the bioavailability problems associated with RIF could be overcome by developing an FDC in which the delivery of the two drugs is segregated, with RIF released in the stomach and INH in the small intestine [90]. A FDC multiparticulate oral system, which boasts ease of manufacture and directly attacks RIF bioavailability concerns and poor patient compliance with existing FDC anti-TB formulations, is yet to be globally developed.

## The rationale for the development of a novel fixed dose combination anti-TB drug delivery system

Drug delivery, which takes into consideration the carrier, the route and the target, has evolved into a strategy of processes or devices designed to enhance the efficacy of therapeutic agents through modified or controlled release. This may involve enhanced bioavailability, improved therapeutic index, or improved patient acceptance or compliance. Drug delivery has been defined by Flynn [91] as 'the use of whatever means possible, be it chemical, physicochemical or mechanical, to regulate a drug's access rate to the body's central compartment, or in some cases, directly to the involved tissues'. The underlying principle that drug delivery technology can bring both therapeutic and commercial value to health care products has been widely accepted. This has created an intense need for presenting 'old' drugs, such as those encompassed in the anti-TB regimen, in new forms utilising novel modes of delivery and dosage forms [92].

Patient failure to take the prescribed medications at the required intervals results in significant morbidity and mortality. The need for research into an oral anti-TB drug delivery system is thus warranted as the efficacy of the current regimen may be improved if the delivery rate, biodegradation, and site-specific targeting can be predicted, monitored, and controlled. From a financial and a global health care perspective, finding new ways to administer the anti-TB drugs in oral form and delivering the multiple-dose, long-term therapy in inexpensive, potent, forms with improved bioavailability is needed. The provision of an administration method, embodied by a dosage form that addresses FDC bioavailability concerns, that will allow patients to safely treat themselves and enhance their compliance with the anti-TB regimen is a significant health care development, particularly in developing countries where access to doctors, clean syringes, sterile needles, and sophisticated treatments are few and far between.

As mentioned, the major route of drug administration is through the oral cavity. This route provides the greatest comfort and convenience of dosing. In addition to avoiding the patient discomfort associated with the parenteral route, the accidental overdosing of the drug can be corrected by withdrawing the unabsorbed drug from the stomach. An anti-TB dosage form that can be orally administered once daily would be optimal for patient compliance [41].

In addressing oral bioavailabiliity concerns, chemical modification or prodrug formation may well be successfully implemented to alter the pharmacokinetics of RIF and INH. Prodrug strategies have successfully improved the oral bioavailability of numerous compounds. In many cases, this involves masking a polar group by esterification to increase lipophilicity and enhance the extent of absorption from the gastrointestinal tract. After absorption, the ester is enzymatically hydrolysed to release the parent drug.

RIF was developed in the Dow-Lepetit Research Laboratories (Milan, Italy) as part of an extensive program of chemical modification of the rifamycins, the natural metabolites of *Nocardia mediterranei *as the hydrazone with N-amino-N'-methylpiperazine that was the most active in the oral treatment of infections in animals and, after successful clinical trials, was introduced into therapeutic use in 1968 [93]. To date, no form of RIF has been widely clinically applied that significantly improves on its oral bioavailability. There is little solubility advantage associated with polymorphic forms, which is inconsequential from a clinical and regulatory point of view [94]. A piperine composition for the improvement of gastrointestinal absorption and systemic utilisation of nutrients and nutritional supplements comprising an extract from the fruit of *Piper *containing a minimum of 98% of pure alkaloid piperine, has been added to multi-drug formulations for the treatment of TB and leprosy. A formulation, containing RIF, INH and PYZ and the said composition, has been tested in human volunteers (Indian Patent No. 1232/DEL/89). In the majority of cases, the comparative levels and peak concentration of the drugs in the presence of piperine were higher. The applicability of these results to bioavailability enhancement, which aims to lower dosage levels and shorten the treatment course, is apparent, but presently cost prohibitive in developing countries [95].

INH was synthesised in 1912 from ethyl isonicotinate and hydrazine by Meyer and Malley as part of their doctoral work in Prague. In 1945, its anti-TB properties were elucidated when nicotinamide was discovered to have anti-TB effects. Being a pro-drug itself, activated through endogenous mycobacterial catalysis, various additional chemical miodifications have been invesigated to alter INH pharmacokinetics. Gianolla et al. [96] attached various acyl groups to the amine (-NH_2_) function of INH for improved lipophilicity and afforded good yields in pro-drugs, which were characterised by spectroscopic and analytical methods. Crooks et al. [97] proposed the fabrication of an INH prodrug through the formation of covalent conjugates of INH with mono-di- and polyoxaalkanoic or thiaalkanoic acids. The conjugation is purported to provide covalent compounds having a chemotherapeutic effect, with enhanced permeation of biological membranes, which remain intact until enzymatically cleaved. As reported, Zhou et al. [68] developed an ionizable prodrug of INH, INHMS, for sustained delivery of INH to alveolar macrophages. The charged prodrug was ion-paired with two different hydrophobic cations: tetrapentylammonium-and tetraheptylammonium-bromide. The prodrug required loading into microparticles for realisation of the targeted sustained effect.

Prodrug formation is clinically relevant in altering *in vivo *disposition kinetics and in attaining sustained release, but developments have not necessarily addressed the deleterious RIF-INH interaction upon oral administration.

Shishoo et al. [80] have promoted the need for the development of a stable formulation containing the RIF-INH combination for differentiated GI release and have suggested enteric-coated tablets or alternative multilayered dosage forms.

In developing an oral modified-release system, cognisance must be taken of the increase in popularity of multiparticulate solid dosage forms (e.g. microparticles, nanoparticles, beads, pellets, granules) in the area of oral controlled drug delivery [98]. Formulation of an anti-TB dosage form as an oral multiparticulate drug-delivery system would furnish many biopharmaceutical advantages when compared with solid single-unit dosage forms in terms of a more even and predictable distribution and transportation in the gastrointestinal tract that is fairly independent of the nutritional state, predictable gastrointestinal transit time, less localised gastrointestinal disturbances and greater product safety; as well as having an application in the improvement of patient compliance. In view of the many benefits offered by multiple unit dosage forms, it is speculated that such systems are particularly useful for site-specific targeting within the gastrointestinal tract [99,100].

In order to manufacture an oral system as cheaply and efficiently as possible in developing countries, intrinsic drug delivery principles may be implemented, employing readily available polymeric and other formulatory excipients to segregate the delivery of the pure drugs. Currently under investigation in our laboratories is the development of a pharmaceutical RIF-INH multiparticulate composition intended for facilitated oral administration that ensures differentiated release of RIF and INH in the gastrointestinal tract. Agrawal et al. [94] have indicated that RIF release in the acidic medium is critical for RIF bioavailability. There is thus the requisite to deliver RIF in a highly available form that will be readily absorbed from the gastric environment. The intended delivery system exemplifies the requirements of small intestinal INH delivery and immediate gastric availability of RIF.

## Conclusion

The current novel drug delivery approaches reviewed all demonstrate significant merit, however, a FDC oral multiparticulate system, which is cheap to manufacture and directly addresses issues of unacceptable RIF bioavailability in FDC anti-TB formulations, is yet to be globally developed The fabrication of a polymeric once-daily oral multiparticulate FDC of the principal anti-TB drugs, which attains segregated delivery of RIF and INH for improved RIF bioavailability, could be a step in the right direction in addressing issues of treatment failure due to patient non-compliance, the consequences of which are devastating.
